# Addressing violence against transgender people in Bangladesh: A call for policy intervention

**DOI:** 10.3389/fsoc.2022.995448

**Published:** 2023-01-30

**Authors:** Arup Barua, Shehreen Ataur Khan

**Affiliations:** ^1^Public Policy, Central European University, Vienna, Austria; ^2^Institut Barcelona d'Estudis Internacionals (IBEI), Barcelona, Spain; ^3^Department of English, Jagannath University, Dhaka, Bangladesh

**Keywords:** violence against transgender people, transgender rights, social exclusion, social policy, policy intervention

## Abstract

Violence, oppression, and cruelty are as old as human civilization itself. Human identity is multi-layered, and deviation from a specific identity may elicit violence, deprivation, and prejudice in various settings. In many countries and societies, the transgender community is one of the most vulnerable groups due to their gender incongruence. Deeply ingrained cultural norms, beliefs, social ignorance, and practices have transferred violence against transgender people over generations, preventing them from enjoying their fundamental human rights. Key objectives of this article are 2-fold: first, this article focuses on violence against transgender people and rights violation in Bangladesh; and second, the report examines the types of violence against the transgender population and the actors who must be involved in providing a solution. Moreover, this article unravels the current organizational and institutional advances in supporting the welfare and rights of the transgender community in Bangladesh. This article concludes that the absence of a dedicated national policy for the protection and welfare of the transgender population impedes the implementation of necessary measures, which should be facilitated by formulation of an appropriate policy, followed by effective implementation.

## Setting the context

Since the dawn of ancient human civilization, violence and atrocities have been committed against individuals, communities, clans, and races by other individuals and groups in order to acquire control. The forms and manifestations of violence have evolved in numerous ways over the years. Globally, the identity of human beings remains a fundamental predictor of their exposure to violence. In *Violence, Identity, and Poverty*, Sen (2008) distinguishes two sets of explanations for the pervasiveness of violence: a cultural approach, and a political economy of poverty and inequality approach. According to the cultural approach, violence results from adversarial collective identities.

Globally, gender identity and sexual orientation are viewed as identifiers, leading to abuses of fundamental human rights, many types of violence, such as denial of access to education, healthcare, housing, employment, and even denial of human dignity. State authority is also inculpated for conducting acts of violence against some quarters of the population based on their identity, rather than respecting, safeguarding, and upholding fundamental human rights. O'Flaherty and Fisher (2008) assert that there are at least eight countries where the death sentence is applied for consensual same-sex acts.

In this article, by adopting a secondary method, we aim to analyze a comprehensive picture of violence against the transgender population in Bangladesh, identify types of violence against them, and highlight the accompanying repercussions. Based on the literature review, which includes journal articles, government policy papers, and media reports, this article provides a stakeholder analysis and evaluates the legal frameworks and barriers to addressing the difficulties.

## Transgender people: Who are they?

Kessler and McKenna noted that individuals are assigned to a particular gender category at birth, typically based on their genitals; however, in regular social encounters, gender is attributed based on other signifiers (Ekins and King, 2006). The contentious nature of the operational definition of the term “transgender” is one of the greatest obstacles associated with working on transgender issues. In addition, transgender beliefs vary between geographic locations since sociocultural and religious norms of a region robustly influence people's perceptions of and behavior toward gender identities.

The definition of a transgender person challenges the common notion of “gender binary,” according to which humans are either female or male. In general, a transgender person refers to an individual who experiences or perceives a gender identity that is ideally distinct from his/her assigned sex at birth. On the basis of biological characteristics, transgender persons cannot be always classified as either men or women.

Stryker (1994) provides a precise definition of the term “transgender”. She attributes its origins to the 1970s, when the term “transgender” “was first developed as a noun by individuals who refused categorization as either transvestites or transsexuals, and who used the phrase to express their own identity”. There are various categories of transgender people: transgender, male to female (MtF); transgender, female to male (FtM); transgender, not female or male, transvestite; and transsexual. Transsexuals “express their identities by a physical change of embodiment, (while) transgender do so through a non-corporeal change” in their gender expression, which is more complicated than altering clothes (ibid).

Stryker continued by elaborating two transgender-related concepts. This article employs (Stryker, 1994, p. 251) second concept, which is given as follows:

“… an umbrella term that refers to all identities or practices that cross over, cut across, move between, or otherwise queer socially constructed sex/gender boundaries. The term includes, but is not limited to, transsexuality, heterosexual transvestism…or the Indian Hijra”.

Prior to the invention of the “transgender” terminology, transsexualism and transvestism terms were in use since the mid-1970s (Ekins and King, 2006). The term “gender identity disorder” on top of the terminology “gender dysphoria”, coined by Fisk in 1973, was frequently used by medical professionals till the 1980s (in Ekins and King, 2006, p. 29).

Many transgender people have another collective identity known as the “hijra” community in South Asian region. Although transgender and hijra are used interchangeably in public discourse in Bangladesh, the term hijra cannot be classified as “with one single category” (Snigdha, 2021; p. 88).

## Transgender people in Bangladesh: From a harsh reality toward aspiration

In Bangladesh, the transgender population is one of the most impoverished and marginalized groups due to their gender identity and sexual orientation. Considering the position of the transgender people, it is evident that their human rights are inadequately recognized in Bangladesh. To our knowledge, no scientific research has been undertaken on this group; thus, there are no reliable data on the transgender population, although they are captured incoherently by various national surveys. Furthermore, this group has been largely ignored in all aspects of national planning and development. According to a 2016 address by the State Minister of Social Welfare, there were ~10,000 transgender people in Bangladesh (BDNews24., 2016). However, some organizations continue to assert that there are roughly a half-million transgender people in the country (Anam, 2015).

The struggle of a transgender person in contemporary Bangladesh begins from birth. In the majority of cases, families often abandon their transgender children when their identity is publicly exposed. Families that do not disown their transgender children, on the other hand, are unwilling to ensure their basic rights, such as the right to education or to play with other children, for the fear of being ostracized and humiliated. As a result, the children are confronted with disparities from the beginning of their lives, which worsen in their later lives. Although such events occur frequently, there are no official statistics on the number of the transgender children who run away in Bangladesh. Furthermore, dropping out of traditional schools/colleges owing to social stigma against the transgender population is an additional factor that needs to be taken with forethought, in order to provide young transgender students with quality education and develop them into a skilled human resource.

## Recent progress on the transgender rights in Bangladesh

On 11 November 2013, the government of Bangladesh recognized transgender as a “third gender,” despite a long history of sociocultural taboos and persistent denial of rights (First Post, 2013; Anam, 2015; Antara, 2018). This was one of the most significant policy decisions made by the administration. This policy decision is viewed as the beginning of a wave of development to promote transgender human rights. In an interview with an electronic media outlet in 2015, the then minister of social welfare stated that beginning in the 2013–14 fiscal year, the government initiated working toward the betterment of the transgender welfare in 21 districts (Jugantor, 2015). In 2014–2015, 14 additional districts were included into transgender social safety net programs. Several non-governmental organizations, primarily sponsored by Western donors, conducted a scattershot of transgender development-related initiatives.

Furthermore, the National Election Commission introduced “third gender” as a third option for selecting a voter's sex on the voter registration form in 2017 by modifying the Voter List Act 2009 and the Voter List Regulations 2012 (Mushtaq, 2018). In the same year, another historic event occurred with the nomination of Nadira Begum, a transgender person with a university degree, in the Rangpur City Council election, a local-level election. Her candidacy and campaign signaled a new attitude shift among the general populace. In an interview with a local media outlet, she stated, “I drew hope from Prime Minister Sheik Hasina's acknowledgment of the hijra community in 2013”, as reported by The Diplomat, indicating that the recognition of the transgender as the third gender has been a revolutionary governmental move (Mushtaq, 2018).

Later, in the early 2019, eight members of the transgender community ran for 50 reserved seats for women in the national parliament, using the ballot of the ruling party. This enthusiasm in competing for positions in the central and national political institutions inspired other transgender people to exercise their civic rights.

Bangladesh Bank, the country's central bank, also enhanced the empowerment and social protection of the transgender community. Bangladesh Bank directed all scheduled banks and financial institutions in the country for corporate social responsibility (CSR) expenditures and oversight *via* its regulatory frameworks, in particular through the publication of a circular by its Sustainable Finance Department in 2014 (Bangladesh Bank, 2014). It suggested a variety of areas to include in CSR expenditures. An allocation for the transgender welfare was not included in the original circular, but its Green Banking and CSR Department emphasized it in the second circular published in June 2015 to include transgender community's welfare (Bangladesh Bank, 2015a). In addition, in June 2015, the Small and Medium Enterprise (SME) and Special Department of the central bank published another circular recognizing the transgender community for financial inclusion in SME, among other disadvantaged communities (Bangladesh Bank, 2015b).

Moreover, in his budget address for the 2021–22 Financial Year (FY), the Finance Minister of Bangladesh proposed a conditional tax benefit for firms who provide work for the “third gender”. Employers must hire at least 10% of their overall staff or 100 transgender workers in order to qualify for a special tax rebate provision, according to his statement (The Ministry of Finance, 2021). However, local human rights organizations and activities voiced their displeasure—a similar clause had been considered previously, so this is nothing new—but nothing noteworthy occurred in practice (The Daily Star, 2021).

In addition to the government, the business sector and private foundations have stepped up to set notable precedents in Bangladesh. These ground-breaking endeavors will transform contemporary societal norms and prejudices against the transgender population. In 2020, for instance, a private charity established Dawatul Koran Third Sex Madrasha, the first madrasha (religious school for Muslims) for the transgender students at Kamrangirchar in Dhaka. The late founder Ahmad Ferdous Bari Chowdhury envisioned this challenging but courageous enterprise (Chowdhury, 2020).

Tasnuva Anan, a transwoman, was hired as a news anchor for a private satellite station in Bangladesh for the first time in 2021, in conjunction with all these procedures (Dhaka Tribune, 2021). The emergence of Anan as a transwoman in Bangladeshi mainstream media has sparked a conversation about visibility. Her experiences of hardship and fight against discrimination uncovered a number of social and psychological disparities against the transgender community in Bangladesh.

Furthermore, a local supermarket chain, Shwapno, hired a group of transgender as salespersons at its several locations across the country (Dhaka Tribune, 2020). Pathao, an online food delivery business, has also employed 50 transgender as delivery personnel in Dhaka. Prior to this recruiting, these individuals received training for their capacity development (The Business Standard, 2021a). Primeasia University, a private university in Bangladesh, for the first time in 2021 announced a full scholarship and tuition waiver for socially disadvantaged groups, including transgender students, in the sphere of higher education (The Business Standard, 2021b).

However, if such progress and visibility are not supported at sociopolitical levels, it may result in disjointed outcomes. To progress toward a sustainable policy for the inclusion of the transgender people in the mainstream of society, discrimination and violence posed against them must be dealt with prudently. The following section provides a detailed analysis of violence suffered by the transgender community in Bangladesh.

## Typologies of violence against transgender community: Experience in Bangladesh

Despite the prevalence of violence against the transgender community in Bangladesh, there are no institutional structure dedicated to tracking this abuse. Even scientific and academic efforts to systemically capture such violence are lacking, and no government data on violence against the transgender population are available. In order to pursue legal actions and evidence-based policy advocacy, there is a growing need to maintain formal documentation and a national database.

Violence against transgender people could be categorized in a variety of ways based on various units of measurement, including actors, causes, and effects. In Bangladesh, transgender people experience a variety of common forms of violence, including structural or systemic violence, physical violence, emotional abuse, and economic violence. In *Violence against transgender people: A review of United States data*, Stotzer (2009) describes three types of violence: sexual violence, physical violence and abuse, and harassment, verbal abuse, and other forms of non-physical violence. He also reaffirms that violence against transgender people begins at an early age.

In Bangladesh, societal and cultural norms, taboos, and behaviors represent unintentional and deeply established systemic violence against transgender people. In the majority of instances, transgender people are often denied access to fundamental rights, including early schooling. In turn, these people are unable to develop the skills necessary to become a productive workforce/human capital and seek acceptable employment. Economic violence can be partially explained by structural violence, in which transgender persons are excluded from many formal economic activities, and co-workers are less likely to appreciate and welcome their presence. At both local and macro-levels, the state is yet to be fully capable of protecting their rights and adopting appropriate interventions.

In addition to structural violence, there are countless cases of transgender people in Bangladesh falling prey to emotional assault. Emotional violence comprises verbal abuse, harassment, and hate crimes against the transgender community. Frequently, transgender people face hate crimes, abuses, and harassment as a result of social standards, a lack of awareness about gender flexibility, and preconceived notions about them. Due to the paucity of employment prospects for the transgender community, some individuals are forced into sex work, where they are routinely exploited and tormented. The rate of suicide is also significantly greater among transgender people, particularly teens (Rapaport, 2018; Toomey et al., 2018). Access to medical healthcare services remains a major obstacle as the transgender are frequently denied medical care and health treatments due to their gender fluid identity.

Following this, to reflect on national statistics, the 2014 census of slum regions and floating population reveals that 4.10% of the transgender population has some type of disability (Bangladesh Bureau of Statistics, 2015). The rate of literacy among the transgender slum dwellers is 31.50%. Ironically, the polio immunization rate among the transgender slum dwellers is significantly lower than that of their male and female counterparts.

**Figure 1:**
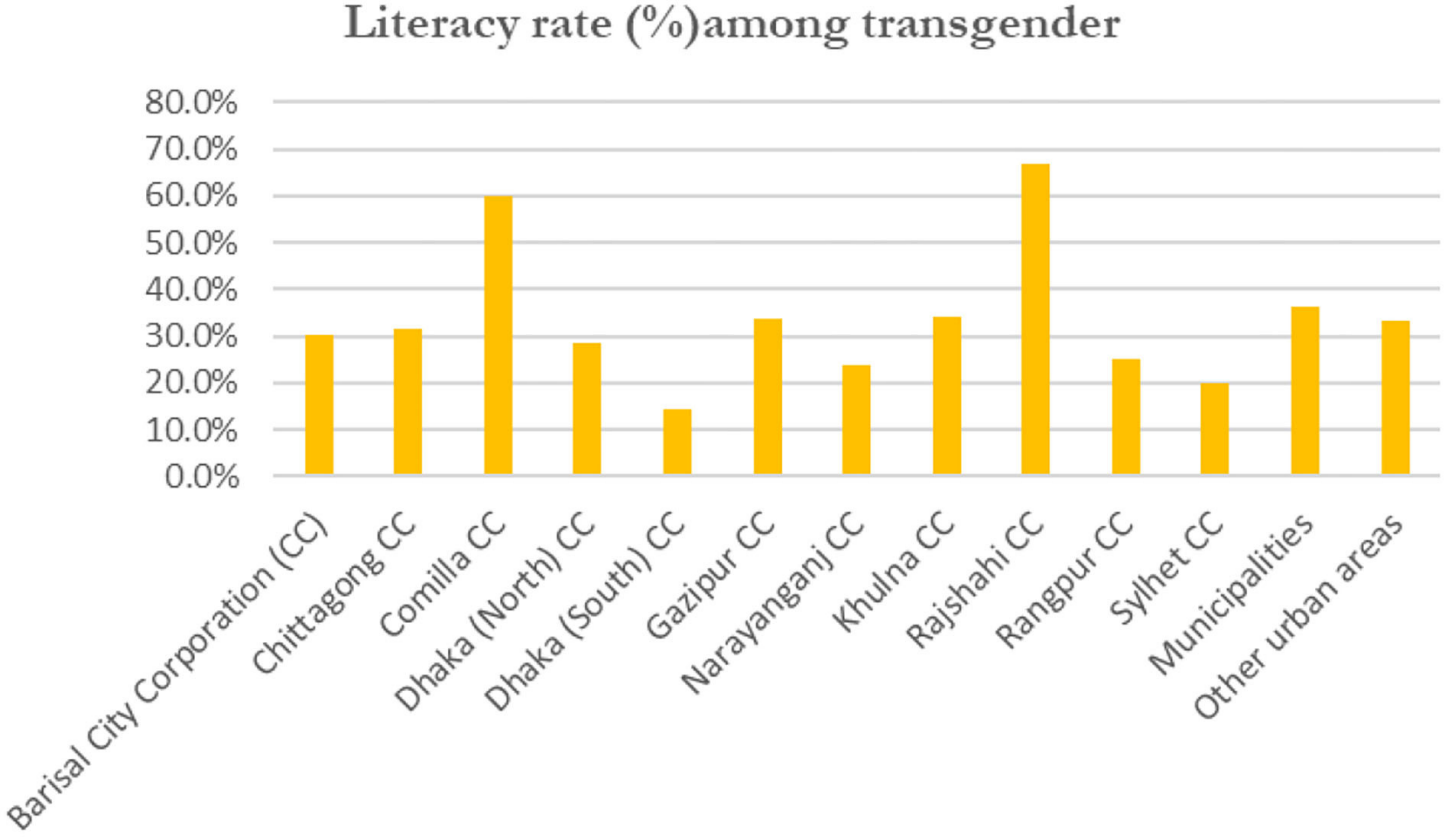
Literacy rate (7 years and older) among transgender slum dwellers (data source Bangladesh Bureau of Statistics, 2015).

Figure 1 compares the literacy rates of transgender residing in various regions of Bangladesh, particularly city corporation areas. The literacy rates of transgender residents in Rajshahi and Comilla municipal corporations are 66.6 and 60%, respectively. By contrast, residents of Dhaka (South) City Corporation have the lowest literacy rate at 14%, followed by Sylhet City Corporation at 20%.

In Bangladesh, transgender persons also encounter discrimination and harassment in the healthcare sector. They are frequently denied access to medical facilities and mental healthcare due to prevalent biases against transgender communities (Human Rights Watch, 2018). A survey conducted in 2019 by the Bandhu Social Welfare Society found that 90% of transgender people who seek healthcare experience mental and sexual harassment. A total of 88% of respondents indicated that their sexual and reproductive healthcare services are inadequate. Along with these disparities, 82% of respondents were concerned about not having access to either women's or men's wards in any hospital, women feel uncomfortable in the presence of the transgender people, while men have a tendency to sexually harass them. A total of 73% of respondents in the same study reported having no access to public health services (Jahan, 2021a).

In Bangladesh, initially there were no specific immunization measures for the transgender community during the COVID-19 pandemic. Consequently, they faced discrepancies in the vaccination campaign. Several articles published in several national newspapers on this topic focus on the inequitable healthcare system. Due to their earlier painful experience with the healthcare system, the majority of the transgender people avoided getting registered for immunization (Jahin, 2021). Moreover, people who have registered experienced difficulties and delays in receiving immunization because their gender identities have not yet been changed in the national database of identity cards, despite the recognition of the transgender as a “third gender” in 2013 (Jahan, 2021b).

The following section provides a structured analysis of the existing scenario regarding the socioeconomic advancement of the transgender community in Bangladesh.

## Stakeholder analysis

This segment aims to map out all relevant stakeholders involved in violence against the transgender population, their rights, and welfare, and potential key actors to be engaged to deal with the issue sustainably.

The analysis of stakeholders was conducted using the aforementioned stakeholder mapping (Table 1). This exercise has identified three groups of stakeholders: primary, secondary, and tertiary based on each actor's motivation, institutional authority, and power to influence the policymaking process and bodies. Primary stakeholders include the transgender community and appropriate ministries in order to sustainably improve the situation with the necessary actions. In addition, parliament, lawmakers, and the judiciary are important stakeholders in the formulation of new policies and enforcement of law. Among these actors, some have greater attention but limited influence. For example, the Ministry of Social Welfare (MoSW) is the essential ministry to lead the welfare activities for the transgender population, but it is constrained by a number of institutional constraints, especially with regard to influencing the national policy framework. The MoSW requires further assistance from the Ministry of Planning in order to implement a large-scale policy reform or formulation. The Bangladesh Bureau of Statistics (BBS) designs and produces national surveys as it does for other national study concerns. Consequently, despite a high level of interest, the MoSW is inadequately equipped to investigate the state of the transgender population and provide appropriate interventions for future. Incorporating transgender-disaggregated data into the national census might be a monumental step for evidence-based planning, which the BBS could advance.

**Table 1:** Stakeholder mapping (developed by authors).

**Typology of stakeholder**	**Actors**	**Interest**	**Decision making and influencing power**
Primary			
	Transgender community	High	Low
	Ministry of Planning	Medium	High
	Ministry of Social Welfare	High	Medium
	Ministry of Finance	Medium	High
	Ministry of Information	Low	High
	Parliament	Low	High
	Elected local representatives	Low	Medium
	Court	Low	High
	Law enforcing agencies (i.e., police)	Medium	High
Secondary	Common citizen	Low	Medium
	Development organizations	Medium	Low
	Development partners	High	Medium
	Private sector/ business association	Low	High
	CSOs, NGOs (working on transgender rights)	High	Low
Tertiary	Media	Low	Medium
	CSOs (in general)	Low	Low

In addition, the parliament and the members of the parliament (MPs) have the legal authority to formulate policy instruments to improve the status of transgender people. This entails the elimination of barriers to accessing education, health services, right to work, and other key concerns that may contribute to their empowerment and integration into society. Since the transgender community represents such a small portion of the total population and thus cannot be a deciding factor in national elections, MPs have relatively little concern over this issue. Therefore, the transgender welfare agenda is frequently on the periphery of legislators' concerns and ultimately fails to make a national agenda. However, parliamentarians can play a vital role through initiating discussions on the transgender welfare in the parliamentary caucus and working groups.

In turn, only development organizations or non-governmental groups focusing on human rights concerns or transgender welfare express interest. However, their activities and lobbying initiatives are frequently dependent completely on donor money. Small-scale groups created by the transgender community have inadequate technical expertise and financial resources to influence central-level decision-makers.

Tertiary-level actors, such as the media, private sector, and business groups, do not exhibit a fervent interest in transgender issues; their proactive actions could influence corporate norms and practices. In Bangladesh, the private sector is the primary source of employment; therefore, the private sector can create new opportunities for transgender persons as productive workers. There are ample options to convert these unskilled/semi-skilled individuals into skilled workers, employable in a variety of areas. Providing education and other skill enhancement programs could be a viable option for their economic empowerment and the promotion of a good life in order to ensure their long-term survival.

## Existing legal instruments and international human rights frameworks

Despite an intricate and terrible state, no national policy or legal instrument addresses the protection and wellbeing of the transgender population in a due manner yet. The Article 19 of the Constitution of Bangladesh, which is recognized as the supreme legislation of the country, ratifies equal opportunity for all citizens: “The State should strive to ensure equal opportunity for all citizens” (Ministry of Law, Parliament and Justice Affairs, 2018, p. 7). Multiple international conventions within human rights frameworks, such as the Universal Declaration of Human Rights, underscore the obligation to protect the human rights and dignity of all individuals, regardless of their identification (United Nations, 1948). In addition, according to the Article 2 of the International Covenant on Civil and Political Rights, state parties must respect and protect the rights of all individuals, regardless of their identification, including their sexual identity (United Nations Human Rights Office of the High Commissioner, 1966). Current global development agenda—sustainable development goals (SDGs) goal 16—depicts the promotion of a peaceful and inclusive society for sustainable development, as well as the provision of access to justice for all (United Nations, 2016). In addition, leaving no one behind is one of the central development slogans of the SDGs.

For the social protection and empowerment of the transgender community and for maximizing their contributions to national development, the formulation of an inclusive and comprehensive policy framework is imperative. Without adopting such instrument, it would not be possible to steer a sustainable social transformation and tolerant society in which the active participation of the general population is essential.

## Conclusion

As a result of various identities, human beings experience a variety of violence. Throughout various stages of their lives, the forms of violence also fluctuate. In this article, we claim that the lack of cohesion in institutional norms, such as a comprehensive national policy and programmatic interventions, hinders dealing with violence against the transgender community in Bangladesh. In Bangladesh, prevailing social norms, attitudes, and practices hinder the wellbeing of the transgender community due to their gender fluid identity. In general, the socialization process of humans has a significant role in sustaining social structures; hence, the introduction of national policy instruments alone would not yield a change at the grassroots level.

In this article, we have also highlighted that a lack of data on the transgender population negatively impacts decision-making at the national and local levels. Existing national censuses and surveys are insufficient to reveal transgender population statistics regarding their access to basic rights, violence, social protection, and empowerment. Without scientific and systematic recording of various forms of violence and deprivation, it is impractical to conceive of a long-term solution to a deeply embedded and socially ingrained problem. Defining a well-structured technique and developing a logical database could be the first step in analyzing and prioritizing transgender needs in all sectors. In addition, a stand-alone integrated policy on the welfare and protection of the transgender population would be essential as well as its effective implementation to foster a national- and/or sub-national-level coordination with all other key actors, including the government, the private sector, and civil society, to form an inclusive and pluralistic society where transgender people with different identities are welcomed and respected.

## Author contributions

AB and SK planned and designed the methodology. AB prepared the draft manuscript. SK reviewed and rewrote the draft manuscript. All authors contributed to the article and approved the submitted version.
